# MicroRNA *mir-34* provides robustness to environmental stress response via the DAF-16 network in *C. elegans*

**DOI:** 10.1038/srep36766

**Published:** 2016-12-01

**Authors:** Meltem Isik, T. Keith Blackwell, Eugene Berezikov

**Affiliations:** 1 Hubrecht Institute-KNAW and University Medical Center Utrecht, Utrecht, The Netherlands; 2 Joslin Diabetes Center, Harvard Stem Cell Institute and Harvard Medical School Department of Genetics, Boston, Massachusetts, United States of America; 3 European Research Institute for the Biology of Ageing, University of Groningen, University Medical Center Groningen, Groningen, The Netherlands

## Abstract

Diverse stresses and aging alter expression levels of microRNAs, suggesting a role for these posttranscriptional regulators of gene expression in stress modulation and longevity. Earlier studies demonstrated a central role for the miR-34 family in promoting cell cycle arrest and cell death following stress in human cells. However, the biological significance of this response was unclear. Here we show that in *C. elegans mir-34* upregulation is necessary for developmental arrest, correct morphogenesis, and adaptation to a lower metabolic state to protect animals against stress-related damage. Either deletion or overexpression of *mir-34* lead to an impaired stress response, which can largely be explained by perturbations in DAF-16/FOXO target gene expression. We demonstrate that *mir-34* expression is regulated by the insulin signaling pathway via a negative feedback loop between miR-34 and DAF-16/FOXO. We propose that *mir-34* provides robustness to stress response programs by controlling noise in the DAF-16/FOXO-regulated gene network.

During severe and long term stress conditions, an altered metabolic state accompanied by structural modification of tissues is a necessary adaptation that provides organisms with a greater chance of survival. Recent studies have shown that microRNAs (miRNAs), which are a class of ~22 nucleotide short non-coding RNAs, play key roles in mediating stress responses^1^. Although loss of function of many individual miRNAs in flies and worms does not cause detectable phenotypes^2^,^3^,^4^, synthetic phenotypes can develop when these mutant animals are subjected to accompanying genetic mutations, environmental perturbations, or the aging process. For instance, shifts in temperature cause development defects in the eyes of miR-7 mutant flies^5^, cardiac overload leads to decrease in survival of miR-208 mutant mice^6^, and osmotic stress response is impaired in miR-8 mutant zebrafish^7^. Therefore, miRNA mutants that do not have obvious phenotypes under normal conditions may exhibit phenotypic defects in stress conditions, giving clues about their stress related functions inside cells.

*mir-34* is one of the miRNAs that shows elevated expression upon stress conditions, such as starvation, dauer formation and aging in *C. elegans*^8^,^9^,^10^,^11^. Notably, *mir-34* expression is also elevated with age in *Drosophila* and mammals^12^,^13^,^14^. In mammals, the miR-34 family is linked to diverse developmental processes, such as cell-cycle arrest, apoptosis, lipid metabolism, metabolic homeostasis and insulin secretion^15^,^16^,^17^,^18^,^19^,^20^,^21^,^22^,^23^,^24^. Two feedback inhibition loops that involve p53/mir-34a/SIRT1 and MK5/Myc/FOXO3a/mir-34b/c have been elucidated and shown to be involved in regulating cellular proliferation in mammals. In *Drosophila* and zebrafish, *mir-34* is maternally inherited and plays a role in the development of the neuronal system^14^,^25^. Furthermore, *mir-34* loss triggers a gene profile of accelerated brain aging and decline in survival, while *mir-34* upregulation extends median lifespan and mitigates neurodegeneration induced by a pathogenic human polyglutamine disease protein^14^. In *Caenorhabditis elegans* loss-of-function mutations in the *mir-34* gene have an abnormal cellular survival response to radiation; these animals are highly radiosensitive in the soma and radioresistant in the germline, as determined by these tissues’ innate mode of cell death post-irradiation^26^.

Despite the growing body of evidence supporting the hypothesis that miR-34 family members (miR-34a, miR-34b, and miR-34c) are important tumor suppressors and mediators of p53 function, mice carrying targeted deletion of the entire miR-34 family do not display increased susceptibility to spontaneous, irradiation-induced, or c-Myc–initiated tumorigenesis^27^. In this study, we used *C. elegans* as a model organism to investigate the effects of *mir-34* mutation and overexpression on the phenotype and transcriptome of animals. We determined that upregulation of *mir-34* under stress conditions and in dauers is necessary to induce a stress related gene expression state that enhances survival. For this purpose, *mir-34* expression is upregulated by DAF-16 – the main downstream transcription factor of the insulin signaling pathway, and its cofactor PQM-1. In turn, miR-34 targets functional insulin signaling receptor-dependent pathways, which are necessary for regulation of growth, correct morphogenesis of tissues and induction of a low metabolic state to allocate energy resources for preserving tissue functions under stress conditions. Furthermore, *mir-34* expression is regulated by a negative feedback loop; consistent with our observations that both mutation and overexpression of *mir-34* under stress conditions can lead to decreased survival rates in adults and dauers. We propose that *mir-34* is a major regulator of stress response pathways, and that its expression is tightly regulated to control gene expression programs that enhance adaptation to stressful environments.

## Results

### *mir-34* expression is regulated by the dauer larva gene expression program

**Figure 1:**
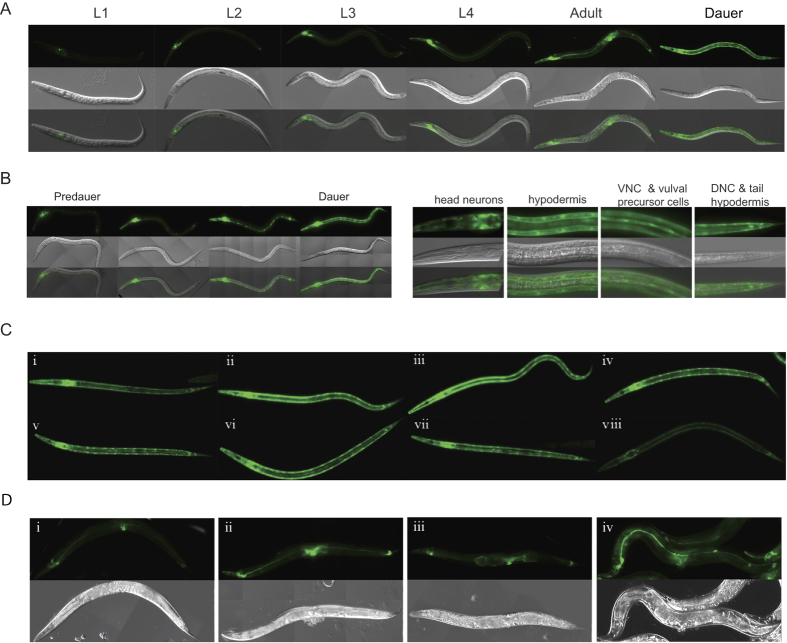
P*mir-34*_*2.2kb*_*::gfp* is expressed in various tissues during development of *C. elegans* and its expression is upregulated in dauers. (**A**) Expression of *Pmir-34*_*2.2kb*_*::gfp* reporter at different stages of animal development. (**B**) Changes in the expression pattern of *Pmir-34*_*2.2kb*_*::gfp* from predauer to dauer stage and detailed expression pattern of *Pmir-34*_*2.2kb*_*::gfp* at dauer stage. (**C**) Elevated P*mir-34*_*2.2kb*_*::gfp* expression is associated with the dauer larva gene expression program. i, WT, ii and iii, insulin-like signaling pathway mutants: *daf-2(e1370*), *pdk-1(sa680*), respectively. iv, v and vi, TGF-β signaling pathway mutants: *daf-1(e1287*), *daf-3(mgDf90*), *daf-7(e1372*). vii, DAF-12/NHR signaling: *daf-9(e1406*). viii, *daf-16(mu86);daf-7(e1372*). (**D**) P*mir-34*_*2.2kb*_*::gfp* expression levels are increase by various stress factors. (i) Three days old animal grown at 20 °C. (ii) One day old animal grown at 25 °C, (iii) Three days old animal starved for two days. (iv) Ten days old animal grown at 20 °C.

To study the relationship between *mir-34,* cell cycle arrest and stress, we focused on the dauer stage of *C. elegans*, which is the stress-resistant diapause stage that forms under harsh environmental conditions such as crowding, high temperatures and starvation^28^,^29^. Stress-induced upregulation of *mir-34* expression was observed in starved worms, dauers and older adults^26^,^31^, which was recapitulated by our P*mir-34*_*2.2kb*_*::gfp* transgenic line (Fig. 1A–D). In predauers, P*mir-34*_*2.2kb*_*::gfp* expression was increased in amphid neurons, especially in AWC neurons. Higher expression levels were later observed in the excretory canal, seam cells and vulval precursor cells, and in ventral and dorsal nerve cord and tail neurons (Fig. 1B). Hypodermal expression appeared in day 1 dauers, and reached the highest level at the 2nd day of the dauer stage, when dauers shed their cuticle. The same expression patterns were observed in dauers of the transgenic P*mir-34*_*5kb*_*::gfp* reporter^26^, suggesting that all crucial regulatory elements for dauer related upregulation of *mir-34* were located within the 2.2 kb upstream region. In mutants that enhance temperature-induced dauer formation, the P*mir-34*_*2.2kb*_*::gfp* transgene expression patterns were similar to those seen in the wild-type (WT) background, implying that high *mir-34* expression derived from differential gene expression at the dauer stage, and not from starvation conditions (Fig. 1C). The highest expression levels were observed with insulin signaling pathway mutant dauers (Fig. 1C ii and iii), suggesting the possibility for the involvement of DAF-16/FOXO in the regulation of *mir-34* expression. Since *daf-16(mu86*) null mutants cannot form dauers, this mutation was introduced into the *daf-7(e1372*);P*mir-34*_*2.2kb*_*::gfp* strain, which is dauer constitutive at 25 °C, in order to investigate whether the high expression of *mir-34* in *daf-7(e1372*) mutant dauers was dependent upon DAF-16. The dauers formed by the *daf-16(mu86);daf-7(e1372*) double mutant had highly diminished reporter expression at dauer stage (Fig. 1C, viii). The same expression pattern was observed with partial dauers of *daf-2(e1370*);*daf-16(mu86*) double mutants, which form with low frequency under stress conditions (data not shown). These results suggest that *mir-34* upregulation is dependent upon DAF-16 in the dauer stage.

### *mir-34* regulates dauer morphogenesis and survival dependent upon the insulin signaling pathway

**Figure 2:**
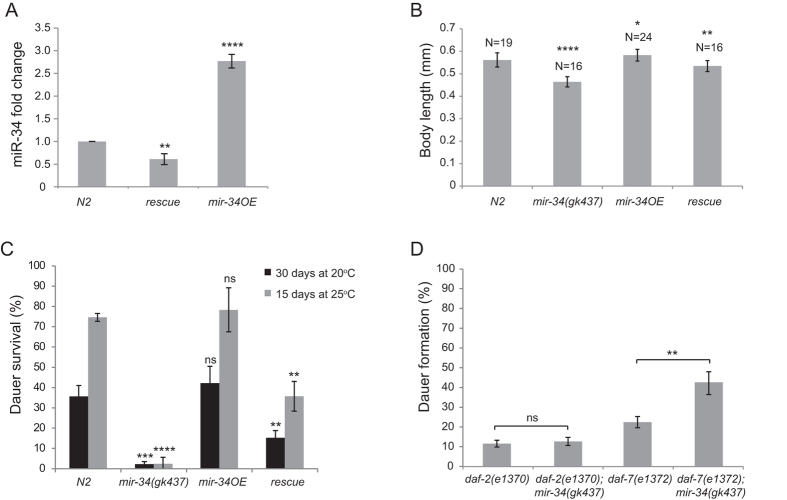
Native levels of *mir-34* expression are required for correct morphogenesis of dauers and dauer survival. (**A**) qPCR confirmation of miR-34 expression levels in the rescue and the overexpression strains. Error bars represent SD. N = 300, **P < 0.01, ***P < 0.0001, unpaired two-tailed *t* test. (**B**) Differences in body sizes of WT, *mir-34(gk437*), *mir-34OE* and rescue strains. Error bars represent SD. *P < 0.05, **P < 0.01, ****P < 0.0001, unpaired two-tailed *t* test. (**C**) Differences in dauer survival of *mir-34(gk437*) and *mir-34OE* dauers at 20 °C and 25 °C. Error bars represent SD. 250 animals are scored per experiment, 3 replicate experiments per condition. Statistical significance of differences between N2 and other genetic backgrounds at a given temperature is calculated using unpaired two-tailed *t* test. **P < 0.01, ***P < 0.001, ****P < 0.0001. (**D**) Differences in dauer formation rates of *mir-34(gk437*) dauers in *daf-2(e1370*) and *daf-7(e1372*) backgrounds. Error bars represent SD. 250 animals were assayed per condition, experiments were performed in triplicate. Statistical significance is calculated by two-tailed unpaired *t* test. **P < 0.01.

We investigated the role of *mir-34* upregulation in dauers by studying dauer morphogenesis and survival in *mir-34* mutants. Around 80% (250 dauers tested) of *mir-34(gk437*) dauers that were selected from starved plates had locomotion defects, and were rolling along their body axis. 80% (50 dauers tested) of these dauers showed alae defects and bulges in their hypodermis (Fig. S1). A *mir-34* rescue strain, which has a single copy insertion of *mir-34* and restores expression of miR-34 to 70% of the wild type level (Fig. 2A), as well as a *mir-34* overexpression strain (*mir-34*OE), which has 4 copies of *mir-34* and expresses miR-34 3-fold higher than wild type (Fig. 2A), rescue these morphological defects (Fig. S1). Additionally, compared to WT dauers, *mir-34(gk437*) mutants had a shorter body size, and worms that overexpress *mir-34* had a slightly longer body size (Fig. 2B). Furthermore, *mir-34(gk437*) mutant dauers exhibited a lower survival rate (at both 20 °C and 25 °C) than WT dauers (Fig. 2C). These dauer body length and survival phenotypes were partially rescued in the rescue strain (Fig. 2C). Intriguingly, the rates of the locomotion phenotype were significantly different in *daf-2(e1370);mir-34(gk437*) dauers compared to *daf-2(e1370*) dauers: rolling was observed in 75% of *daf-2(e1370*) dauers and in 95% of *daf-2(e1370);mir-34(gk437*) dauers (250 animals tested in each condition, *P* < 10^−6^, chi-square test). At the same time, there was no significant difference between *daf-2(e1370*) and *daf-2(e1370);mir-34(gk437*) worms in terms of body size and survival (data not shown). Additionally, the *mir-34(gk437*) mutation enhanced dauer formation in *daf-7(e1372*) mutant background but it did not have an effect on dauer formation in *daf-2(e1370*) mutants (Fig. 2D). We conclude that *mir-34* upregulation is necessary for inducing developmental arrest with correct morphogenesis and enhanced survival of dauers, and that this role of *mir-34* relies on a functional insulin signaling receptor, DAF-2.

### *mir-34* is regulated by DAF-16, PQM-1 and DAF-12

**Figure 3:**
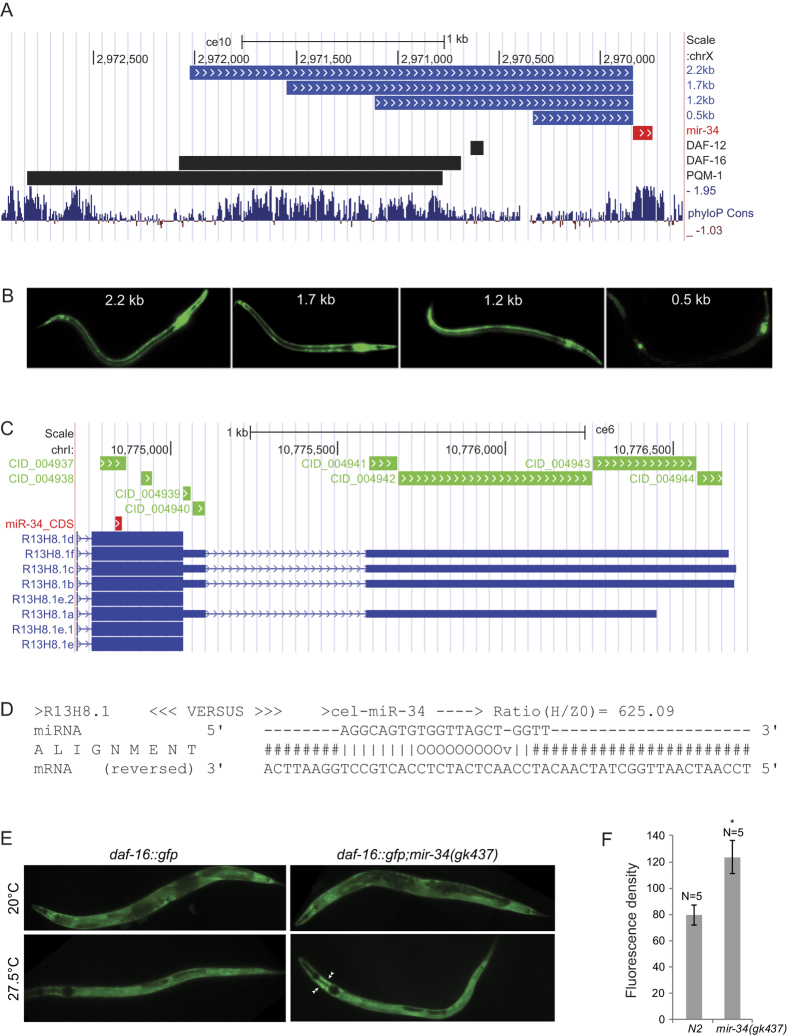
*mir-34* is regulated by DAF-16 and targets *daf-16*. (**A**) Upstream region of *mir-34* pre-miRNA (in red) showing sequences used as promoters in P*mir34::gfp* strains in blue and location of DAF-12, DAF-16 and PQM-1 modENCODE ChIP-seq peaks in black. (**B**) Expression patters of P*mir34::gfp* strains with different promoter length. (**C**) Last exon and 3′UTR of *daf-16* transcript (in blue), location of AGO-CLIP regions (in green) from Grosswendt *et al*.^39^ and highly-scoring MIRZA miR-34 target prediction (in red). (**D**). Detailed MIRZA alignment of the miR-34 target in *daf-16* mRNA shown in panel C. (**E**) Expression of DAF-16::GFP is elevated with temperature in amphid neurons (indicated by arrows) in *mir-34(gk437*) mutants but not in wild-type animals. (**F**) Quantification of fluorescence density of DAF-16::GFP in wild-type and *mir-34(gk437*) background at 27.5 °C. Data are expressed in arbitrary fluorescence units. Error bars represent SD. Statistical significance is calculated by two-tailed unpaired *t* test. *P < 0.05.

The insulin signaling pathway regulates dauer-related phenotypes and responses to stress conditions by regulating nuclear localization of its downstream target transcription factor, DAF-16/FOXO^32^,^33^. PQM-1 complements DAF-16 by directly binding to the DAF-16-associated element (DAE), and balancing developmental and stress response programs^34^. We identified the minimal promoter region responsible for *mir-34* upregulation by generating several P*mir-34*_*2.2kb*_*::gfp* strains with shorter upstream regions relative to the initial 2.2 kb promoter (Fig. 3A). The analysis of these strains indicated that sequences between 0.5 kb and 1.2 kb upstream of *mir-34* gene are essential for its regulation (Fig. 3B). According to ChIP-seq data available from modENCODE^35^, this region is bound among others by the DAF-16, PQM-1 and DAF-12 transcription factors (Fig. 3A). Small internal promoter deletions in the region bound by these TFs revealed that DAF-12 binding elements, insulin response element (IRE) and GA-repeats were required for *mir-34* expression in hypodermis and seam cells (Fig. S2), and DAF-16 was necessary for activation of P*mir-34*_*2.2kb*_*::gfp* expression in dauers (Fig. 1C and Fig. S3A) and in amphid neurons, especially AWC neurons of adults (Fig. S3D). P*mir-34*_*2.2kb*_*::gfp* levels were similar to WT levels in *daf-2(e1370);daf-16(mu86*) background (Fig. S3D), suggesting that other factors were also involved in *mir-34* induction upon inhibition of insulin signaling pathway. Furthermore, analysis of P*mir-34*_*2.2kb*_*::gfp* in excretory gland cells in various mutant background showed a direct correlation between DAF-16 levels and reporter expression (Fig. S3B,C). In contrast to its effect in AWC neurons, DAF-16 was required for P*mir-34*_*2.2kb*_*::gfp* induction in excretory gland cells only under stress conditions and in sensitized genetic backgrounds, where DAF-16 is active and nuclearly localized^36^,^37^,^38^. Additionally, glucose supplementation reduced P*mir-34*_*2.2kb*_*::gfp* levels (Fig. S3C), and prolonged stress conditions resulted in reduction of P*mir-34*_*2.2kb*_*::gfp* expression in many tissues of the worms.

### miR-34 targets *daf-16*

To understand the molecular programs underlying phenotypic changes observed in the *mir-34* mutant and overexpression strains, we identified experimentally supported targets of miR-34 using Argonaute crosslinking and immunoprecipitation (AGO-CLIP) data generated by Grosswendt *et al*.^39^, in combination with miRNA target predictions calculated by MIRZA software^40^. In total, we identified 1304 genes with a MIRZA score above 100, of which 214 are also supported by AGO-CLIP data (Table S1). The *daf-16* gene was among the top 20 targets that showed the highest MIRZA scores and were supported by AGO-CLIP evidence. The predicted miR-34 target region is located in the last coding exon of *daf-16*, not far from the stop codon (Fig. 3C). It has a MIRZA score of 625, and exhibits perfect complementarity to nucleotides 1–8 of the mature miR-34 sequence (Fig. 3D). The combination of AGO-CLIP evidence and highly-scoring MIRZA prediction suggests that *daf-16* is very likely regulated by miR-34 and suggests existence of a negative feedback-loop between *daf-16* and *mir-34.*

### Transcriptome analysis reveals the crosstalk between DAF-16 and *mir-34*

**Figure 4:**
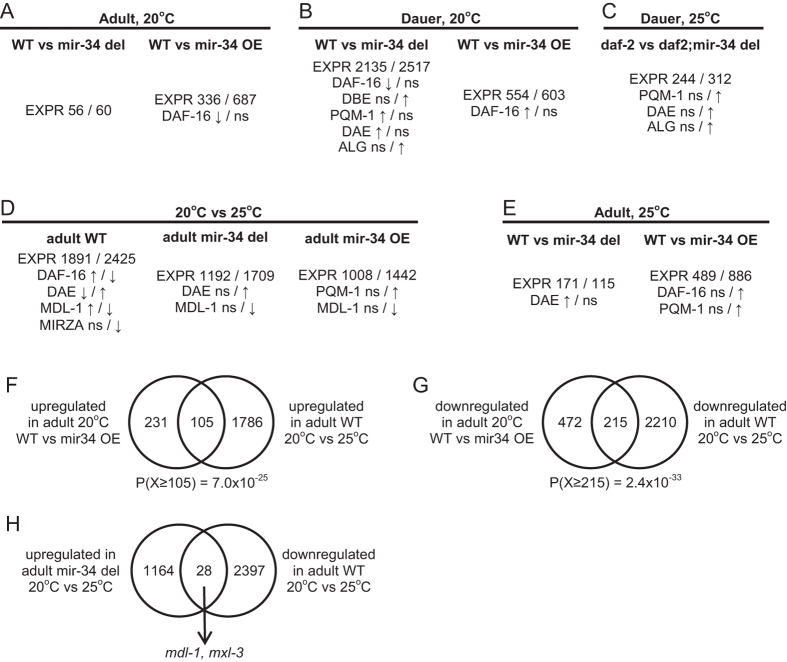
Effects of *mir-34* deletion or overexpression on gene expression under various conditions. (**A–E**) Number of differentially expressed genes (EXPR) and enrichment statistics for several regulatory signals. DAF-16, PQM-1 and MDL-1, occupancy in promoter regions based on modENCODE ChIP-seq data for respective transcription factors. DBE, DAE, presence of the respective sequence motives in the promoter regions. MIRZA, miR-34 target predictions calculated by MIRZA. ALG – presence of AGO-CLIP regions from Grosswendt *et al*.^39^, overlapping miR-34 MIRZA predictions. The first number in each column relates to genes expressed higher in the first conditions, the second – to the genes expressed higher in the second condition. Up- and down- arrows represent significant over- and under-representation respectively. Statistical significance is calculated by Pearson’s Chi-squared test with Yates’ continuity correction and adjusted for multiple comparisons by Bonferroni correction. Adjusted *P* < 0.01 was considered as significant. ns – not significant. (**F,G**) Overexpression of miR-34 at 20 °C shifts gene expression profile in the same direction as temperature stress. (**H**) Genes that behave differently in WT and *mir-34* mutants upon temperature stress.

To investigate the possible crosstalk between *mir-34* and DAF-16, we performed microarray gene expression analysis for several genetic backgrounds and stress conditions (Table S1, Fig. 4). In *mir-34*OE and *mir-34* mutant dauers a large number of genes were differentially expressed compared to WT dauers (1157 and 4652, respectively; Fig. 4B), consistent with the observed phenotypes and upregulated *mir-34* expression pattern in dauers. Genes that were expressed higher in the absence of *mir-34* were significantly enriched for DAF-16 binding elements (DBE) and AGO-CLIP supported miR-34 targets, whereas genes which were expressed higher in the wild-type dauers were significantly depleted for DAF-16 binding but enriched for PQM-1 binding the DAE (Fig. 4B). The DAF-16 enrichment was reversed when expression of *mir-34OE* and WT dauers was compared, although the overall number of differentially expressed genes decreased (Fig. 4B). These data suggest that differential expression of genes between wild-type and mutant dauers is the result of both direct regulation by miR-34 and indirect regulation via DAF-16/PQM-1 binding.

The number of differentially expressed genes was highly diminished in *mir-34(gk437*) mutants when the comparison was done in the *daf-2(e1370*) background, where nuclear DAF-16 levels are saturated (Fig. 4C). This finding was in line with the lower amount of phenotypic changes observed between *daf-2(e1370*) and *daf-2(e1370);mir-34(gk437*) mutant worms.

Earlier studies identified sets of genes that are either up-regulated (class 1) or down-regulated (class 2) in long-lived *daf-2* mutants^41^, and genes that are differentially expressed in dauers and non-dauers^42^. We observed a large overlap between class 1 genes, dauer related genes, and genes that were up-regulated in *mir-34(gk437*) dauers, and between class 2 genes, non-dauer genes, and genes that were down-regulated in *mir-34(gk437*) dauers (Fig. S4). As expected, these relationships were reversed for *mir-34OE* dauers. Thus, a strong *daf-2*/dauer transcriptional signature is present in *mir-34(gk437*) mutant dauers, as it is also evident from our transcriptome analysis (Fig. S5A (i, ii)).

GO term analysis of genes that were upregulated in the *mir-34(gk437*) dauer background and had ALG-1 binding sites and/or MIRZA scores higher than 100 revealed upregulation of genes encoding glycoproteins, cytoskeletal genes, intermediate filaments, extracellular matrix proteins, transmembrane and transport proteins (Table S2). Upregulation of *mir-34* in hypodermis and seam cells and the morphological defects of *mir-34(gk437*) mutants correlate with these GO terms. Additionally, there was an upregulation of glycolysis/gluconeogenesis related genes (Table S2), which is in line with the findings that glycolytic and gluconeogenic pathways are upregulated in dauers^43^. Consistent with these *C. elegans* results, RNA-Seq analysis of a published GEO dataset for hippocampus of wild-type adult male C57BL/6 mice^44^ revealed upregulation of genes related to extracellular matrix, cell adhesion, basement membrane and anti-apoptosis when *mir-34* was knocked down by adeno-associated viral (AAV)-delivered mir-34 sponges (180 upregulated and 36 downregulated genes, FDR < 0.01) (Table S3).

### miR-34 expression is necessary for inducing stress response programs

Next, we investigated how temperature stress influences gene expression at the adult stage. Dauer formation and heat stress in adults resulted in highly overlapping gene expression patterns (Fig. S4, S5A (i, iii)), revealing the stress response genes that are commonly regulated under these conditions^45^. While in wild-type animals temperature shift from 20 °C to 25 °C resulted in 1891 and 2425 down- and up- regulated genes respectively (Fig. 4D), the number of differentially expressed genes was smaller in *mir-34(gk437*) (1192/1709 genes) and *mir-34OE* (1008/1442) backgrounds (Fig. 4D). This suggests that precise levels of miR-34 are required to elicit proper response to heat stress, and that deviations from these levels impair the stress response program. Indeed, impaired stress response was observed in *mir-34(gk437*) and *mir-34OE* compared to WT worms at the transcriptome level in adult stage (Fig. S5A (iii, iv, v)).

In line with these results, both *mir-34(gk437*) and *mir-34OE* adults were more sensitive to hypoxia, heat stress, and starvation. The rescue strain partially rescued the phenotypes observed in these assays (Fig. S5B–D).

If miR-34 expression helps in establishing the stress response program, then gene expression changes when *mir-34* is overexpressed under normal conditions should overlap with stress response genes that are observed in WT animals grown under heat stress. Indeed, we observed an overlap of 105 and 215 genes in up- and down-regulated sets, respectively (Fig. 4F,G), and both results are highly statistically significant (*P* < 10^−24^, hypergeometric probability). These results suggest that indeed *mir-34* plays a direct role in establishing the stress response program.

Which genes might be particularly sensitive to miR-34 levels in this stress response program? To address this question, we looked for genes that responded oppositely to heat stress in WT and *mir-34(gk437*) animals. There were only 28 such genes for which expression increased with temperature in WT animals but decreased in *mir-34(gk437*) animals (Fig. 4H), including *mdl-1 and mxl-3. mdl-1* is a basic helix-loop-helix (bHLH) transcription factor that acts as a part of the Myc-like interaction network in *C. elegans. S*imilar to vertebrate MAD, MDL-1 dimerizes with MXL-1 and MXL-3 and plays a role in integrating diverse longevity signals^46^,^47^,^48^. The *mdl-1* gene promoter is bound by DAF-16 and PQM-1, according to modENCODE data^35^, and the *mdl-1* mRNA is targeted by miR-34 according to AGO-CLIP data^39^ and MIRZA prediction, although the MIRZA score is modest (Table S1). This suggests that the *C. elegans myc* network may play an important role in modulating a stress response program downstream of *mir-34*. Indeed, differentially expressed genes from various comparisons appear to exhibit statistically significant under- or over-representation of MDL-1 ChiP-seq binding in their promoters (Fig. 4D).

Other transcription factors, which showed same pattern as *mdl-1* in terms of sensitivity to *mir-34* levels included *nhr-23, egl-13* and *zip-7*. NHR-23 is a critical co-regulator of functionally linked genes involved in growth and molting. EGL-13 is required for maintenance of the uterine pi cell fate and mutations in *egl-13* affect the cell fusion process that makes the vulval-uterine connection and consequently egg laying. These transcription factors may also be responsible for miR-34 dependent transcriptome and phenotypic changes under stress conditions.

### Further evidence for a DAF-16-*mir-34* feedback inhibition loop

Finally, we sought additional evidence for *daf-16* regulation by miR-34 in a *daf-16::gfp* reporter strain and in gene expression data. We observed higher DAF-16::GFP levels in *mir-34(gk437*) mutants grown at high temperatures (*P* = 0.0175, *t* test), accompanied by higher levels of nuclear localization of the translational fusion protein in amphid neurons, however, there were no significant differences under normal growth conditions (Fig. 3E,F). miRNAs mainly act by translational repression of their target mRNAs and may also decrease their levels by destabilization, but the extent of mRNA destabilization may vary^49^,^50^,^51^,^52^,^53^. The levels of *daf-16* mRNA decreased by 12% and 8%, respectively, in adults and dauers overexpressing miR-34 at 20 °C, although the statistical significance of these changes is low (adjusted *P* value 0.41 and 0.58 respectively). At 25 °C, overexpression of miR-34 in adults results in a 17% decrease of *daf-16* levels (adjusted *P* = 0.161). Furthermore, in N2 animals shift from 20 °C to 25 °C does not significantly change *daf-16* levels (6% change, *P* = 0.523) but overexpression of miR-34 at 25 °C leads to a 25% downregulation of *daf-16* compared to WT at 20 °C (*P* = 0.012). Although the observed changes in *daf-16* expression upon miR-34 overexpression are not large, combined with the experimental AGO-CLIP data^39^ and DAF-16::GFP reporter analysis results, they suggest direct regulation of *daf-16* by miR-34.

## Discussion

The biological functions of many miRNAs can only be elucidated in a context-specific manner^1^, suggesting that many miRNAs function in cellular stress responses. For example, *mir-34* deletion mutants do not show any abnormal morphological, developmental or biological phenotypes under standard laboratory culture conditions. However, miR-34 is critical in the DNA damage response in both mammals and *C. elegans*. Additionally, in mammals, *mir-34* expression is transcriptionally regulated by p53 in response to numerous forms of DNA damage^17^,^54^,^55^ and in *C. elegans mir-34* mutants exhibit developmental defects related to cell migrations under stress conditions^56^.

In this study, we demonstrated that miR-34 levels are upregulated to sustain a gene expression program that is associated with morphological and metabolic adaptation of stress. We showed that *mir-34* mutation results in morphogenesis defects of dauers, which correlates with our transcriptome analysis results that shows deregulation of cell adhesion, cytoskeleton, ECM and basement membrane related gene categories both in of *mir-34* mutant dauers and *mir-34* knockdown mouse hippocampus. Although *mir-34* mutant dauers exhibit more a dauer-related transcriptional signature, changes in gene category representation are accompanied by the body defects and short survival rates of *mir-34* mutants. Therefore, we think that upregulation of *mir-34* is necessary for correct morphogenesis of tissues to ensure long survival of dauers.

According to a previous study the autophagy-related mRNA ATG9A was regulated by *mir-34* in mammalian cells^57^. However, we found that in *C. elegans atg-9* mRNA expression was lower in *mir-34* and *daf-2;mir-34* backgrounds, and did not observe a significant change in *atg-9* transcript levels in adult stages (Table S1). Additionally, ATG9A did not show a significant change in expression levels in *mir-34* knockdown in male mouse hippocampus (Table S3). However, several other autophagy-related genes (*lgg-1, atg-13, atg-16.2, unc-51, bec-1*) were downregulated in *mir-34*OE dauers compared to *mir-34* mutants (Table S1), which may suggest autophagy inhibition by *mir-34* as was proposed in the aforementioned study^57^.

Both *miR-34* and *daf-16* expression levels were shown to be increased upon dauer formation^26^,^58^ and also in adults in an age-dependent manner^59^,^60^, suggesting a link between these regulators of gene expression. By engineering promoter truncations we showed that an IRE sequence, which binds DAF-16, is present in *mir-34* promoter. We also observed that upregulation of P*mir-34*_*2.2kb*_*::gfp* in dauers is abolished by mutating this region, and in the *daf-16(mu86*) background. Furthermore, according to our combined analysis of MIRZA scores and AGO-CLIP data, one of the top predicted miR-34 targets is *daf-16/FOXO*. We demonstrated DAF-16 dependent changes in the transcriptomes of animals that lack and overexpress *mir-34*. The survival defect of *mir-34* mutants required a functional insulin signaling pathway, where DAF-16 nuclear localization levels are not saturated, and probably DAF-16 activity is more prone to regulation by *mir-34*. Additionally, *mdl-1 and mxl-3,* from the Myc-like interaction network in *C. elegans*, showed *mir-34* dependent downregulation under high temperature growth, suggesting that the *myc* network is a part of the stress response pathway that is modulated by *daf-16* and *mir-34*. Thus, our results suggest that *mir-34* is involved in a feedback inhibition loop that includes the *daf-16* and *myc* networks to regulate a stress response program in *C. elegans* (Fig. S6). According to this regulatory loop, if miR-34 becomes upregulated above threshold levels, *mir-34* expression is lowered via the feedback inhibition of DAF-16, which results in reduced stress resistance. We observed reduced stress resistance in both *mir-34* mutants and overexpressors, supporting the role of this feedback inhibition loop in regulation of *mir-34* and DAF-16 levels to reduce the fluctuations in *daf-16* and *myc* network target expression levels under stress conditions.

Such a regulatory loop that involves miR-34b/c, FOXO3a and Myc was previously described in mammalian cells^61^. According to the model, MK5 activates *mir-34b/c* expression via phosphorylation of FOXO3a, thereby promoting nuclear localization of FOXO3a and enabling it to induce *mir-34b/c* expression and arrest proliferation. Expression of MK5 in turn is directly activated by Myc, forming a negative feedback loop. In line with these findings, our results suggest that *mir-34* has an evolutionarily conserved function in orchestrating responses to stresses, by modulating expression levels of DAF-16/FOXO and the Myc network. We speculate that the reported increase in *mir-34* mutations in tumors^62^,^63^,^64^ may impair the functioning of this network.

## Methods

### Strains

All *C. elegans* strains (Table S4) were maintained and handled as described previously^65^.

### Construction of GFP reporters

*Mir-34* promoter was selected using UCSC genome browser as a 2.2 kb sequence upstream of *mir-34* precursor sequence and amplified by primers with flanking restriction enzyme sites, AflII and NotI. Amplified sequence was cloned into modified pCFJ151 vector that has *unc-119* gene and *gfp* sequence with *let-858* 3′UTR sequence. Truncated *mir-34* promoter sequences were amplified using primers with flanking AflII and NotI restriction enzyme sites and cloned in the same way. For deletions up to 100 bp we used Quickchange site directed mutagenesis. For longer deletions primers were designed for the amplification of the whole plasmid excluding the region that is desired to be deleted. Transgenic lines were made by microparticle bombardment^66^ of *unc-119(ed3*) animals with *promoter::gfp;unc-119*(+), and the transformants were screened for stable integration. The resulting strains for each construct are listed in Table S4. Sequences of all oligonucleotides used in the study are listed in the Table S5.

### Construction of *mir-34* rescue and overexpression strains

P*mir-34*_*2.2kb*_::*mir-34* was cloned into MosSCI plasmid and isolated plasmid was microinjected into N2 worms together with marker plasmids. Integrated worms were selected from plates and sequenced for the transgene.

### qPCR validation of miR-34 expression

qPCR was performed on N2, *mir-34(gk437*) and N2 and *mir-34(gk437*) worms carrying P*mir-34*_*2.2kb*_::*mir-34* transgene by using TaqMan kit.

### Dauer locomotion assay

Dauers were selected from starved plates by 1% SDS treatment and placed on humid NGM plates with no bacteria. The locomotion of dauers were observed and recorded as rollers and non-rollers.

### Dauer formation assay

Parents raised continuously on food were transferred from 20 °C to plates without food to lay eggs and then removed after 4–6 hr. Plates were incubated at 22 °C for 54 hours for both *daf-2(e1370*) and *daf-7(e1372*) backgrounds and dauer and non-dauer animals were counted. This permitted correct scoring of transient dauers that recover rapidly^67^. Dauers were distinguished by 1% SDS treatment. An average of 300 animals was assayed per condition in triplicate experiments.

### Dauer survival assay

Dauers were selected from a plate that was starved for five days by 1% SDS treatment. Wells of a 24-well plate were filled with 300 μL water and 50 dauers were distributed per well. Plates were incubated at 20 °C and 25 °C for 60 days or 30 days respectively. Dauers were transferred to agar plates and scored for viability by touching.

### Stress assays

For all the stress assays, gravid adult worms of each tested strain were allowed to lay eggs on NGM plates seeded with OP50 for 2–3 h to produce relatively synchronous populations of progeny. All assays were repeated at least three times.

### Heat stress assay

Day 3 adult worms grown on OP50-NGM plates were shifted to 35 °C. Duplicate plates for each strain were scored for each time point. Because the scoring was done at room temperature, once the worms were pulled from 35 °C and scored for survival, they were discarded to avoid the complication of recovery from heat shock during the time of scoring.

### Hypoxic stress assay

Day 3 adult worms grown on OP50-NGM plates were placed in 0.5 mL Eppendorf tubes with M9. The tubes were filled completely with M9 and the lids were closed after making sure that no air bubbles were left inside the tubes. 6–7 tubes (20 worms per tube) were prepared per each strain. Alive worms were counted after 28 hr incubation at 20 °C.

### Starvation stress assay

FUDR treated day 3 adult worms were placed in M9 solution without any bacteria and worms were scored for survival after 7 days of incubation on shaker.

### Oxidative stress assay

Day 3 adult worms grown on OP50-NGM plates were placed in 100 μL of 200 mM paraquat solution in a 96 well plate (25 worms per well). At least 120 worms were used per each strain. Alive worms were counted at 2 hr intervals.

### Imaging and quantification of GFP

To examine changes in expression of GFP reporters in different mutant backgrounds, we picked 20 worms and then measured the level of GFP expression using quantitative fluorescence microscopy. Specifically, we used pixel intensity to quantify the level of GFP expression in WT and mutant backgrounds. Comparison of all images was carried out on the same day with the same microscope settings. Images were analyzed using Adobe Photoshop and ImageJ software. Phenotypic analysis of the worms was done by Nomarski optics.

### Microarray analysis

Microarray data were generated using Agilent single-color platform according to the manufacturer protocols. Total RNA was prepared from three independent biological replicates using TRIzol reagent, DNase treated and purified by Qiagen RNeasy mini kit. RNA labeling, hybridization and microarray processing was performed by the Genomics Core Facility, EMBL, Heidelberg. Results were analyzed using limma package in R^68^.

### RNA-Seq analysis

GEO dataset with accession number GSE68884 was used for the analysis of the effect of AAV-delivered *mir-34* sponges on male mouse hippocampus compared to GFP sponges. HISAT2^69^ was used for the alignment of reads to the mouse genome using the following options: “$HISAT2_HOME/hisat2 -p 25 –phred33 –rna-strandness R –trim5 5 -q -x./Genome_mouse/grcm38_snp_tran/genome_snp_tran -U SRR20253xx.fastq.gz -S SRR20253xx.sam”. Quality control analysis of the data and count reads for EdgeR analysis were obtained by running QoRTs^70^ package in cluster with the following options: “java -jar./QoRTs-master/QoRTs_1.1.8/QoRTs.jar QC –minMAPQ 60–maxReadLength 45 –singleEnded –stranded –seqReadCt xxxxxxxx SRR20253xx.bam /Mus_musculus.GRCm38.83.gtf SRR20253xx_QoRTs/”. EdgeR analysis was done in R using glmLRT method^71^.

### miRNA target predictions

For target predictions, we used a recently published algorithm MIRZA^40^, and next to *C. elegans* genes, we included prediction for human genes. In this algorithm, the whole miRNA sequence is considered, not only 3′UTR, since there is growing evidence for functional targeting in coding sequence^72^,^73^,^74^,^75^,^76^. A good target would have a MIRZA score above 50, and Table S1 also lists how many miR-34 targets were found in a gene, and their cumulative MIRZA score.

### ChiP-seq enrichment analysis

Data for DAF-16, PQM-1, DAF-12, MDL-1 and MML-1 ChIP regions were extracted from cumulative gff3 files downloaded from modENCODE website (datasets modENCODE_591, modENCODE_2623, modENCODE_3381, modENCODE_2601 and modENCODE_2943 respectively). Gene list was filtered to contain only protein coding genes according to WS220 GTF annotation, and promoter regions were defined in the window −300:+100 relative to TSS. ChIP regions overlapping defined promoter regions were counted and enrichment calculations were performed using R.

### Statistical analysis

Statistical analysis was performed using Graphpad Prism 7 and R. Unpaired or paired *t*-tests were used to determine significance in experiments in which only two groups with large sample size and approximately normal distribution were compared.

### GO-term enrichment analysis

GO-term enrichment analysis was performed by using DAVID Bioinformatics Database^77^,^78^.

### Accession numbers

Microarray data presented in this study is available in GEO under accession number GSE76413.

## Additional Information

**How to cite this article**: Isik, M. *et al*. MicroRNA *mir-34* provides robustness to environmental stress response via the DAF-16 network in *C. elegans. Sci. Rep.* **6**, 36766; doi: 10.1038/srep36766 (2016).

**Publisher's note:** Springer Nature remains neutral with regard to jurisdictional claims in published maps and institutional affiliations.

## Supplementary Material

Supplementary Information

Supplementary Table 1

Supplementary Table 2

Supplementary Table 3

## References

[b1] Leung A. K. L. & Sharp P. A. MicroRNA functions in stress responses. Mol. Cell 40, 205–215 (2010). 20965416 10.1016/j.molcel.2010.09.027 PMC2996264

[b2] Bushati N. & Cohen S. M. MicroRNA Functions. Annual Review of Cell and Developmental Biology 23, 175–205 (2007). 10.1146/annurev.cellbio.23.090506.123406 17506695

[b3] Leaman D. . Antisense-mediated depletion reveals essential and specific functions of microRNAs in Drosophila development. Cell 121, 1097–1108 (2005). 15989958 10.1016/j.cell.2005.04.016

[b4] Miska E. A. . Most Caenorhabditis elegans microRNAs Are Individually Not Essential for Development or Viability. Plos Genet 3, e215 (2007). 18085825 10.1371/journal.pgen.0030215 PMC2134938

[b5] Li X., Cassidy J. J., Reinke C. A., Fischboeck S. & Carthew R. W. A microRNA imparts robustness against environmental fluctuation during development. Cell 137, 273–282 (2009). 19379693 10.1016/j.cell.2009.01.058 PMC2674871

[b6] van Rooij E. . Control of stress-dependent cardiac growth and gene expression by a microRNA. Science 316, 575–579 (2007). 17379774 10.1126/science.1139089

[b7] Flynt A. S. . miR-8 microRNAs regulate the response to osmotic stress in zebrafish embryos. J Cell Biol 185, 115–127 (2009). 19332888 10.1083/jcb.200807026 PMC2700511

[b8] Garcia-Segura L. . High-Throughput Profiling of Caenorhabditis elegans Starvation-Responsive microRNAs. Plos One 10, e0142262 (2015). 26554708 10.1371/journal.pone.0142262 PMC4640506

[b9] Ibáñez-Ventoso C. . Modulated microRNA expression during adult lifespan in Caenorhabditis elegans. Aging Cell 5, 235–246 (2006). 16842496 10.1111/j.1474-9726.2006.00210.x

[b10] Karp X., Hammell M., Ow M. C. & Ambros V. Effect of life history on microRNA expression during *C. elegans* development. RNA 17, 639–651 (2011). 21343388 10.1261/rna.2310111 PMC3062175

[b11] de Lencastre A. . MicroRNAs both promote and antagonize longevity in *C. elegans*. Curr. Biol. 20, 2159–2168 (2010). 21129974 10.1016/j.cub.2010.11.015 PMC3023310

[b12] Khanna A., Muthusamy S., Liang R., Sarojini H. & Wang E. Gain of survival signaling by down-regulation of three key miRNAs in brain of calorie-restricted mice. Aging (Albany NY) 3, 223–236 (2011). 21415464 10.18632/aging.100276 PMC3091518

[b13] Li X., Khanna A., Li N. & Wang E. Circulatory miR34a as an RNAbased, noninvasive biomarker for brain aging. Aging (Albany NY) 3, 985–1002 (2011). 22064828 10.18632/aging.100371 PMC3229974

[b14] Liu N. . The microRNA miR-34 modulates ageing and neurodegeneration in Drosophila. Nature 482, 519–523 (2012). 22343898 10.1038/nature10810 PMC3326599

[b15] Achari C., Winslow S., Ceder Y. & Larsson C. Expression of miR-34c induces G2/M cell cycle arrest in breast cancer cells. BMC Cancer 14, (2014). 10.1186/1471-2407-14-538 PMC4125691 25064703

[b16] Chakraborty C., George Priya Doss C. & Bandyopadhyay S. miRNAs in insulin resistance and diabetes-associated pancreatic cancer: the ‘minute and miracle’ molecule moving as a monitor in the ‘genomic galaxy’. Curr Drug Targets 14, 1110–1117 (2013). 23834149 10.2174/13894501113149990182

[b17] Chang T.-C. . Transactivation of miR-34a by p53 Broadly Influences Gene Expression and Promotes Apoptosis. Molecular Cell 26, 745–752 (2007). 17540599 10.1016/j.molcel.2007.05.010 PMC1939978

[b18] Chen F. & Hu S.-J. Effect of microRNA-34a in cell cycle, differentiation, and apoptosis: a review. J. Biochem. Mol. Toxicol. 26, 79–86 (2012). 22162084 10.1002/jbt.20412

[b19] Cole K. A. . A Functional Screen Identifies miR-34a as a Candidate Neuroblastoma Tumor Suppressor Gene. Mol Cancer Res 6, 735–742 (2008). 18505919 10.1158/1541-7786.MCR-07-2102 PMC3760152

[b20] Esguerra J. L. S., Mollet I. G., Salunkhe V. A., Wendt A. & Eliasson L. Regulation of Pancreatic Beta Cell Stimulus-Secretion Coupling by microRNAs. Genes (Basel) 5, 1018–1031 (2014). 25383562 10.3390/genes5041018 PMC4276924

[b21] Misso G. . Mir-34: A New Weapon Against Cancer? Mol Ther Nucleic Acids 3, e194 (2014). 10.1038/mtna.2014.47 PMC4222652 25247240

[b22] Rokavec M., Li H., Jiang L. & Hermeking H. The p53/miR-34 axis in development and disease. J Mol Cell Biol 6, 214–230 (2014). 24815299 10.1093/jmcb/mju003

[b23] Rottiers V. & Näär A. M. MicroRNAs in metabolism and metabolic disorders. Nat Rev Mol Cell Biol 13, 239–250 (2012). 22436747 10.1038/nrm3313 PMC4021399

[b24] Xu Y. . A metabolic stress-inducible miR-34a-HNF4α pathway regulates lipid and lipoprotein metabolism. Nat Commun 6, 7466 (2015). 26100857 10.1038/ncomms8466 PMC4479415

[b25] Soni K. . miR-34 is maternally inherited in Drosophila melanogaster and Danio rerio. Nucleic Acids Res. 41, 4470–4480 (2013). 23470996 10.1093/nar/gkt139 PMC3632126

[b26] Kato M. . The mir-34 microRNA is required for the DNA damage response *in vivo* in *C. elegans* and *in vitro* in human breast cancer cells. Oncogene 28, 2419–2424 (2009). 19421141 10.1038/onc.2009.106 PMC2941141

[b27] Concepcion C. P. . Intact p53-Dependent Responses in miR-34–Deficient Mice. PLoS Genetics 8, e1002797 (2012). 22844244 10.1371/journal.pgen.1002797 PMC3406012

[b28] Murakami S. & Johnson T. E. A Genetic Pathway Conferring Life Extension and Resistance to UV Stress in Caenorhabditis elegans. Genetics 143, 1207–1218 (1996). 8807294 10.1093/genetics/143.3.1207 PMC1207391

[b29] Riddle D. L. & Albert P. S. In C. elegans II (eds. Riddle D. L., Blumenthal T., Meyer B. J. & Priess J. R.) (Cold Spring Harbor Laboratory Press, 1997). 21413221

[b30] Karp X., Hammell M., Ow M. C. & Ambros V. Effect of life history on microRNA expression during *C. elegans* development. RNA 17, 639–651 (2011). 21343388 10.1261/rna.2310111 PMC3062175

[b31] Smith-Vikos T. & Slack F. J. MicroRNAs and their roles in aging. J Cell Sci 125, 7–17 (2012). 22294612 10.1242/jcs.099200 PMC3269020

[b32] Ogg S. . The Fork head transcription factor DAF-16 transduces insulin-like metabolic and longevity signals in *C. elegans*. Nature 389, 994–999 (1997). 9353126 10.1038/40194

[b33] Lin K. daf-16: An HNF-3/forkhead Family Member That Can Function to Double the Life-Span of Caenorhabditis elegans. Science 278, 1319–1322 (1997). 9360933 10.1126/science.278.5341.1319

[b34] Tepper R. G. . PQM-1 Complements DAF-16 as a Key Transcriptional Regulator of DAF-2-Mediated Development and Longevity. Cell 154, 676–690 (2013). 23911329 10.1016/j.cell.2013.07.006 PMC3763726

[b35] Contrino S. . modMine: flexible access to modENCODE data. Nucleic Acids Res 40, D1082–D1088 (2012). 22080565 10.1093/nar/gkr921 PMC3245176

[b36] Henderson S. T. & Johnson T. E. daf-16 integrates developmental and environmental inputs to mediate aging in the nematode Caenorhabditis elegans. Current Biology 11, 1975–1980 (2001). 11747825 10.1016/s0960-9822(01)00594-2

[b37] Wolf M., Nunes F., Henkel A., Heinick A. & Paul R. J. The MAP kinase JNK-1 of Caenorhabditis elegans: Location, activation, and influences over temperature-dependent insulin-like signaling, stress responses, and fitness. J. Cell. Physiol. 214, 721–729 (2008). 17894411 10.1002/jcp.21269

[b38] Leiser S. F., Begun A. & Kaeberlein M. HIF-1 modulates longevity and healthspan in a temperature-dependent manner. Aging Cell 10, 318–326 (2011). 21241450 10.1111/j.1474-9726.2011.00672.x PMC3980873

[b39] Grosswendt S. . Unambiguous identification of miRNA:target site interactions by different types of ligation reactions. Mol. Cell 54, 1042–1054 (2014). 24857550 10.1016/j.molcel.2014.03.049 PMC4181535

[b40] Khorshid M., Hausser J., Zavolan M. & van Nimwegen E. A biophysical miRNA-mRNA interaction model infers canonical and noncanonical targets. Nature Methods 10, 253–255 (2013). 23334102 10.1038/nmeth.2341

[b41] Murphy C. T. . Genes that act downstream of DAF-16 to influence the lifespan of Caenorhabditis elegans. Nature 424, 277–283 (2003). 12845331 10.1038/nature01789

[b42] Jones S. J. . Changes in gene expression associated with developmental arrest and longevity in Caenorhabditis elegans. Genome Res. 11, 1346–1352 (2001). 11483575 10.1101/gr.184401

[b43] Wang J. & Kim S. K. Global analysis of dauer gene expression in Caenorhabditis elegans. Development 130, 1621–1634 (2003). 12620986 10.1242/dev.00363

[b44] Malmevik J. . Distinct cognitive effects and underlying transcriptome changes upon inhibition of individual miRNAs in hippocampal neurons. Scientific Reports 6, 19879 (2016). 26813637 10.1038/srep19879 PMC4728481

[b45] Zhou K. I., Pincus Z. & Slack F. J. Longevity and stress in Caenorhabditis elegans. Aging (Albany NY) 3, 733–753 (2011). 21937765 10.18632/aging.100367 PMC3184976

[b46] Riesen M. . MDL-1, a growth- and tumor-suppressor, slows aging and prevents germline hyperplasia and hypertrophy in *C. elegans*. Aging (Albany NY) 6, 98–117 (2014). 24531613 10.18632/aging.100638 PMC3969279

[b47] Yuan J., Tirabassi R. S., Bush A. B. & Cole M. D. The *C. elegans* MDL-1 and MXL-1 proteins can functionally substitute for vertebrate MAD and MAX. Oncogene 17, 1109–1118 (1998). 9764821 10.1038/sj.onc.1202036

[b48] Johnson D. W. . The Caenorhabditis elegans Myc-Mondo/Mad complexes integrate diverse longevity signals. PLoS Genet. 10, e1004278 (2014). 24699255 10.1371/journal.pgen.1004278 PMC3974684

[b49] Hausser J. & Zavolan M. Identification and consequences of miRNA-target interactions — beyond repression of gene expression. Nat Rev Genet 15, 599–612 (2014). 25022902 10.1038/nrg3765

[b50] Selbach M. . Widespread changes in protein synthesis induced by microRNAs. Nature 455, 58–63 (2008). 18668040 10.1038/nature07228

[b51] Baek D. . The impact of microRNAs on protein output. Nature 455, 64–71 (2008). 18668037 10.1038/nature07242 PMC2745094

[b52] Djuranovic S., Nahvi A. & Green R. miRNA-Mediated Gene Silencing by Translational Repression Followed by mRNA Deadenylation and Decay. Science 336, 237–240 (2012). 22499947 10.1126/science.1215691 PMC3971879

[b53] Bazzini A. A., Lee M. T. & Giraldez A. J. Ribosome Profiling Shows That miR-430 Reduces Translation Before Causing mRNA Decay in Zebrafish. Science 336, 233–237 (2012). 22422859 10.1126/science.1215704 PMC3547538

[b54] He L. . A microRNA component of the p53 tumour suppressor network. Nature 447, 1130–1134 (2007). 17554337 10.1038/nature05939 PMC4590999

[b55] Raver-Shapira N. . Transcriptional Activation of miR-34a Contributes to p53-Mediated Apoptosis. Molecular Cell 26, 731–743 (2007). 17540598 10.1016/j.molcel.2007.05.017

[b56] Burke S. L., Hammell M. & Ambros V. Robust Distal Tip Cell Pathfinding in the Face of Temperature Stress Is Ensured by Two Conserved microRNAS in Caenorhabditis elegans. Genetics 200, 1201–1218 (2015). 26078280 10.1534/genetics.115.179184 PMC4574240

[b57] Yang J. . MiR-34 modulates Caenorhabditis elegans lifespan via repressing the autophagy gene atg9. AGE 35, 11–22 (2013). 22081425 10.1007/s11357-011-9324-3 PMC3543738

[b58] Lee R. Y. N., Hench J. & Ruvkun G. Regulation of *C. elegans* DAF-16 and its human ortholog FKHRL1 by the daf-2 insulin-like signaling pathway. Current Biology 11, 1950–1957 (2001). 11747821 10.1016/s0960-9822(01)00595-4

[b59] de Lencastre A. . MicroRNAs Both Promote and Antagonize Longevity in *C. elegans*. Current Biology 20, 2159–2168 (2010). 21129974 10.1016/j.cub.2010.11.015 PMC3023310

[b60] Bansal A. . Transcriptional regulation of Caenorhabditis elegans FOXO/DAF-16 modulates lifespan. Longevity & Healthspan 3, 5 (2014). 24834345 10.1186/2046-2395-3-5 PMC4022319

[b61] Kress T. R. . The MK5/PRAK kinase and Myc form a negative feedback loop that is disrupted during colorectal tumorigenesis. Mol. Cell 41, 445–457 (2011). 21329882 10.1016/j.molcel.2011.01.023

[b62] Bader A. G. miR-34 – a microRNA replacement therapy is headed to the clinic. Frontiers in Genetics 3 (2012). 10.3389/fgene.2012.00120 PMC3387671 22783274

[b63] Hermeking H. MicroRNAs in the p53 network: micromanagement of tumour suppression. Nat. Rev. Cancer 12, 613–626 (2012). 22898542 10.1038/nrc3318

[b64] Henrich K.-O., Schwab M. & Westermann F. 1p36 tumor suppression–a matter of dosage? Cancer Res. 72, 6079–6088 (2012). 23172308 10.1158/0008-5472.CAN-12-2230

[b65] Brenner S. The Genetics of Caenorhabditis Elegans. Genetics 77, 71–94 (1974). 4366476 10.1093/genetics/77.1.71 PMC1213120

[b66] Isik M. & Berezikov E. Biolistic transformation of Caenorhabditis elegans. Methods Mol. Biol. 940, 77–86 (2013). 23104335 10.1007/978-1-62703-110-3_7

[b67] Ailion M. & Thomas J. H. Dauer formation induced by high temperatures in Caenorhabditis elegans. Genetics 156, 1047–1067 (2000). 11063684 10.1093/genetics/156.3.1047 PMC1461313

[b68] Ritchie M. E. . limma powers differential expression analyses for RNA-sequencing and microarray studies. Nucleic Acids Research 43, e47–e47 (2015). 25605792 10.1093/nar/gkv007 PMC4402510

[b69] Kim D., Langmead B. & Salzberg S. L. HISAT: a fast spliced aligner with low memory requirements. Nature Methods 12, 357–360 (2015). 25751142 10.1038/nmeth.3317 PMC4655817

[b70] Hartley S. W. & Mullikin J. C. QoRTs: a comprehensive toolset for quality control and data processing of RNA-Seq experiments. BMC Bioinformatics 16 (2015). 10.1186/s12859-015-0670-5 PMC4506620 26187896

[b71] Robinson M. D., McCarthy D. J. & Smyth G. K. edgeR: a Bioconductor package for differential expression analysis of digital gene expression data. Bioinformatics 26, 139–140 (2010). 19910308 10.1093/bioinformatics/btp616 PMC2796818

[b72] Forman J. J., Legesse-Miller A. & Coller H. A. A search for conserved sequences in coding regions reveals that the let-7 microRNA targets Dicer within its coding sequence. Proceedings of the National Academy of Sciences 105, 14879–14884 (2008). 10.1073/pnas.0803230105 PMC2567461 18812516

[b73] Qin W. . miR-24 Regulates Apoptosis by Targeting the Open Reading Frame (ORF) Region of FAF1 in Cancer Cells. Plos One 5, e9429 (2010). 20195546 10.1371/journal.pone.0009429 PMC2828487

[b74] Ott C. E. . MicroRNAs differentially expressed in postnatal aortic development downregulate elastin via 3′ UTR and coding-sequence binding sites. PLoS ONE 6, e16250 (2011). 21305018 10.1371/journal.pone.0016250 PMC3031556

[b75] Huang F. W. D., Qin J., Reidys C. M. & Stadler P. F. Target prediction and a statistical sampling algorithm for RNA-RNA interaction. Bioinformatics 26, 175–181 (2010). 19910305 10.1093/bioinformatics/btp635 PMC2804298

[b76] Hausser J., Syed A. P., Bilen B. & Zavolan M. Analysis of CDS-located miRNA target sites suggests that they can effectively inhibit translation. Genome Research 23, 604–615 (2013). 23335364 10.1101/gr.139758.112 PMC3613578

[b77] Huang D. W., Sherman B. T. & Lempicki R. A. Bioinformatics enrichment tools: paths toward the comprehensive functional analysis of large gene lists. Nucleic Acids Research 37, 1–13 (2009). 19033363 10.1093/nar/gkn923 PMC2615629

[b78] Huang D. W., Sherman B. T. & Lempicki R. A. Systematic and integrative analysis of large gene lists using DAVID bioinformatics resources. Nature Protocols 4, 44–57 (2008). 10.1038/nprot.2008.211 19131956

