# Supplementary material for: MicroRNA mir-34 provides robustness to environmental stress response via the DAF-16 network in C. elegans
Source: Sci Rep. 2016 Dec 1;6:36766. doi: 10.1038/srep36766 (PMC5131338; doi:10.1038/srep36766)
Supplement: Supplementary Information [file srep36766-s1.pdf]

# MicroRNA *mir-34* provides robustness to environmental stress response via the DAF-16 network in *C. elegans*

Meltem Isik[1,2], T. Keith Blackwell[2], and Eugene Berezikov[1,3]

[1]Hubrecht Institute-KNAW and University Medical Center Utrecht, Utrecht, The Netherlands; [2]Joslin Diabetes Center, Harvard Stem Cell Institute, and Harvard Medical School Department of Genetics, Boston, Massachusetts, United States of America; [3]European Research Institute for the Biology of Ageing, University of Groningen, University Medical Center Groningen, Groningen, The Netherlands.

## Supplementary Figures and Tables:

**Supplementary Figure S1.** Morphological defects in *mir-34(gk437)* dauers.
**Supplementary Figure S2.** Effect of insulin response element (IRE), DAF-12, GA-repeat deletions on P*mir-34*$_{2.2kb}$*::gfp* expression in dauers.
**Supplementary Figure S3. P*mir-34*$_{2.2kb}$*::gfp* expression is regulated by DAF-16 in both adult and dauer stages.**
**Supplementary Figure S4.** Gene overlap analysis for up- and down-regulated genes for conditions tested in microarray analysis, class 1, class 2, dauer and non-dauer genes.
**Supplementary Figure S5.** Both *mir-34(gk437)* and *mir-34OE* result in impaired stress response.
**Supplementary Figure S6.** Model for daf-16/mir-34 feedback inhibition loop in regulating dauer morphogenesis and survival and environmental stress response.

**Supplementary Table S4.** *C. elegans* strains used in the study.
**Supplementary Table S5.** Sequences of oligonucleotides used in the study.

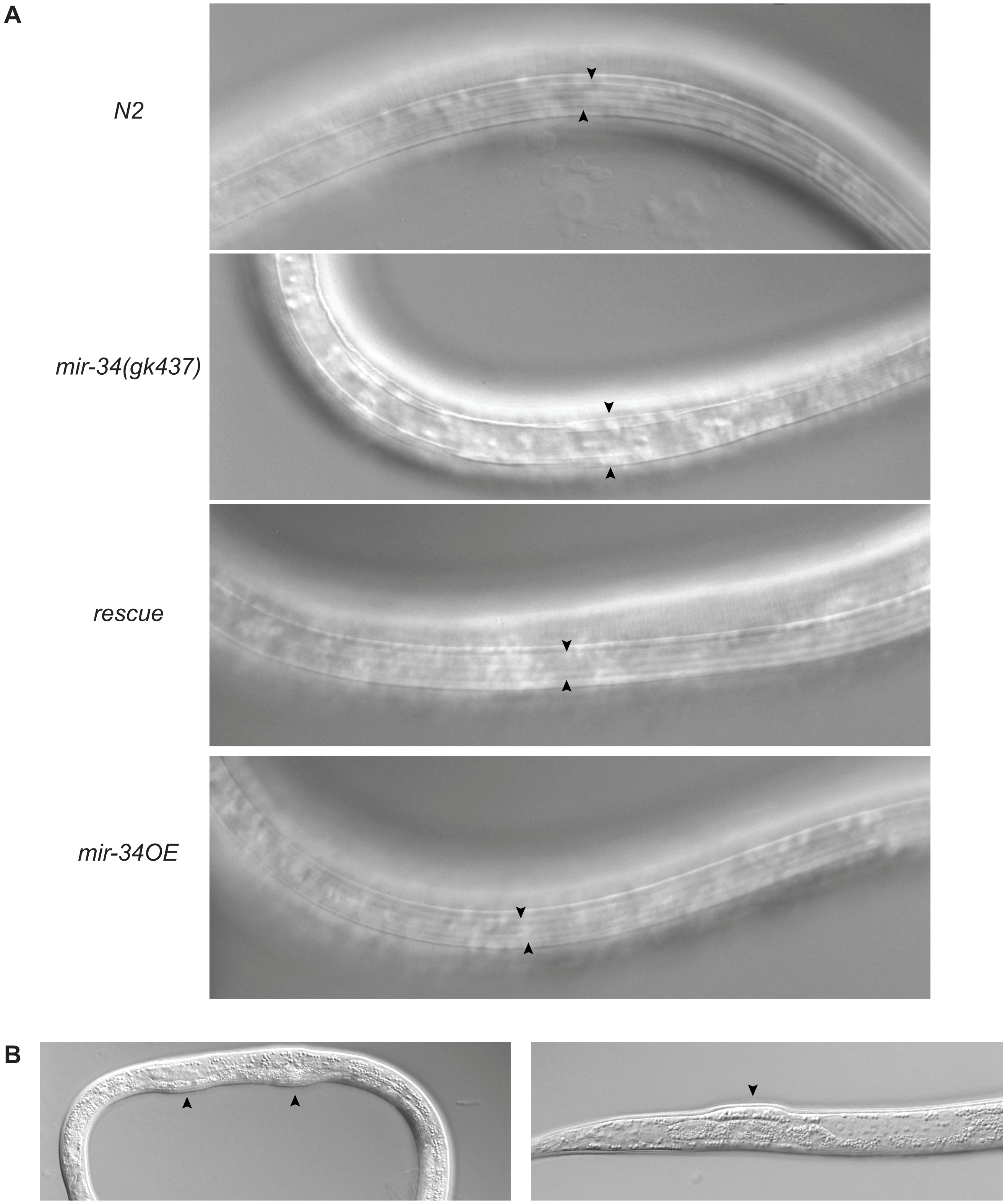


**Supplementary Figure S1. Morphological defects in *mir-34(gk437)* dauers.** (**A**) Defects in alae formation. Alae positions are indicated by arrows. Alae are formed by single protruding ridges in wild-type animals, and it is split into several ridges in *mir-34(gk437)* dauers. The defect is rescued in the rescue and overexpression strains. (**B**) Defects in body shapes due to bulges in hypodermis (indicated by arrows).

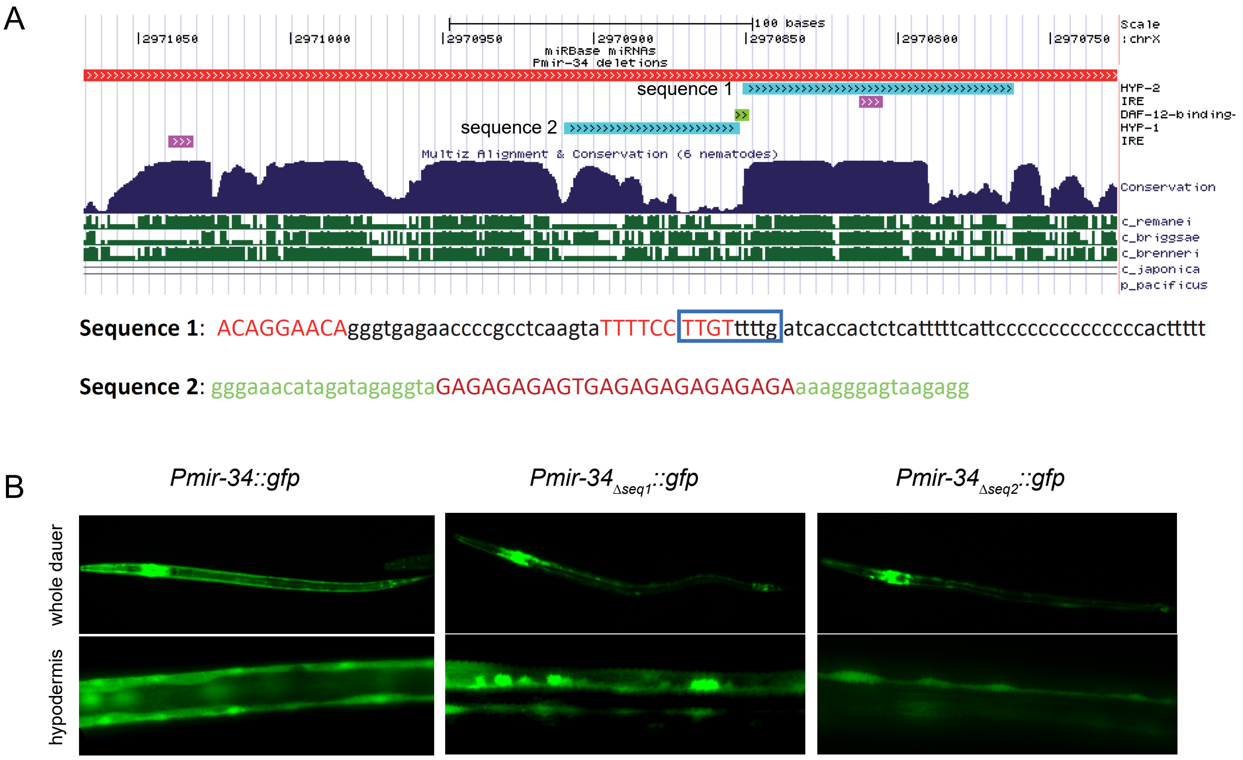


**Supplementary Figure S2. Effect of insulin response element (IRE), DAF-12, GA-repeat deletions on P*mir-34*$_{2.2kb}$*::gfp* expression in dauers.** (**A**) Location and sequence of tested deletions. (**B**) Hypodermal expression of P*mir-34*$_{2.2kb}$*::gfp* is diminished in dauers when sequence 1 (DAF-12 binding element and IRE) is deleted from *mir-34* promoter. Both hypodermal and seam cell P*mir34::gfp* expression in dauers is lost when GA-repeat elements are deleted from *mir-34* promoter.

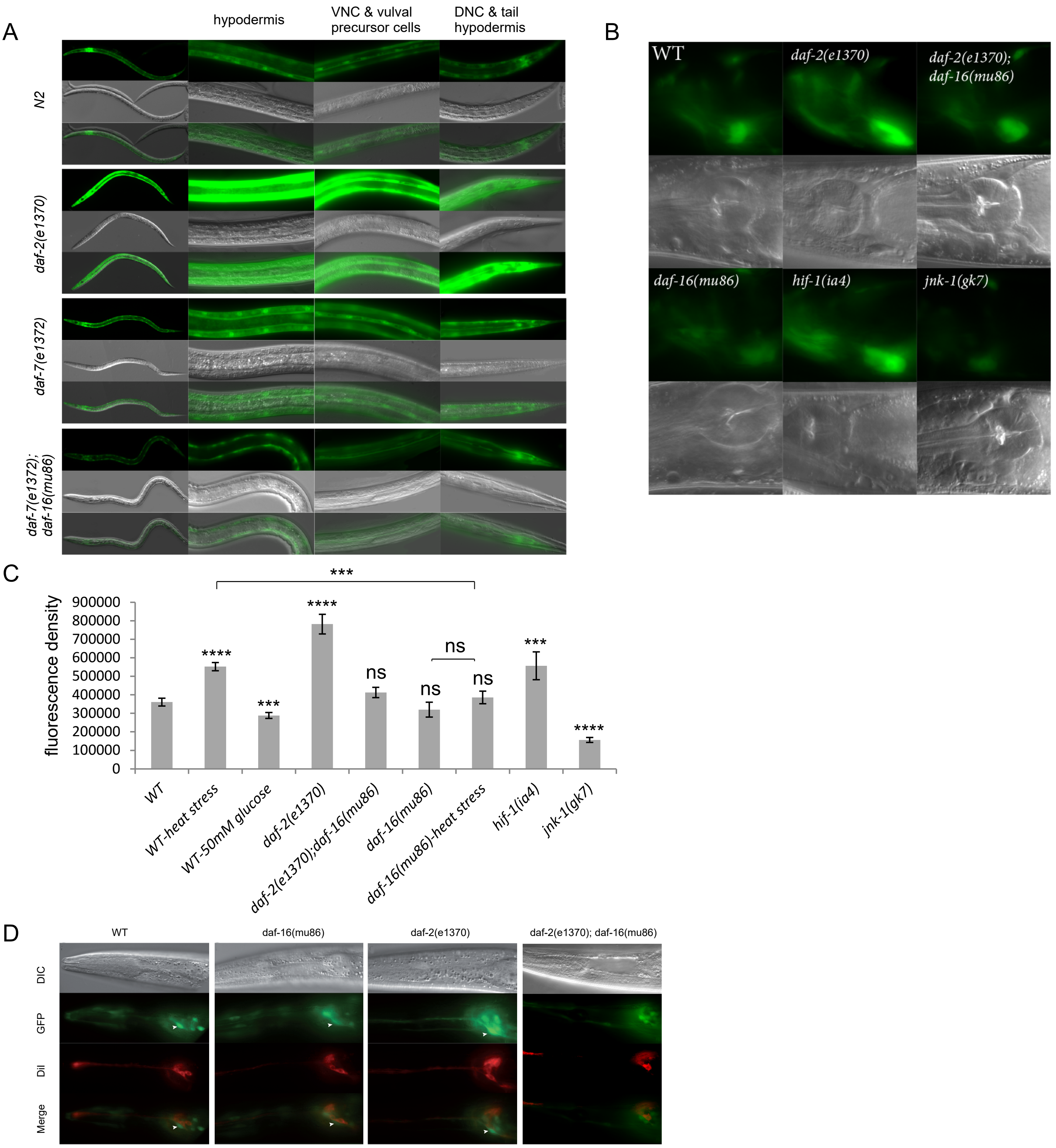


**Supplementary Figure S3. P*mir-34*$_{2.2kb}$*::gfp* expression is regulated by DAF-16 in both adult and dauer stages.** (**A**) DAF-16 regulates *mir-34* expression in dauers. (**B**) P*mir-34*$_{2.2kb}$*::gfp* expression levels in excretory gland cells of (i) WT, (ii) *daf-2(e1370),* (iii) *daf-2(e1370);daf-16(mu86),* (iv) *daf-16(mu86),* (v) *hif-1(ia4) and* (vi) *jnk-1(gk7) backgrounds* (**C**) Quantification of fluorescence density of P*mir-34*$_{2.2kb}$::GFP in various genetic backgrounds, under heat stress and glucose supplementation. Data are expressed in arbitrary fluorescence units. Error bars represent SD. Statistical significance is calculated by two-tailed unpaired *t* test. ***$P < 0.001$, ****$P < 0.0001$. (**D**) DAF-16 is required for *mir-34* expression in AWC amphid neurons of adults.

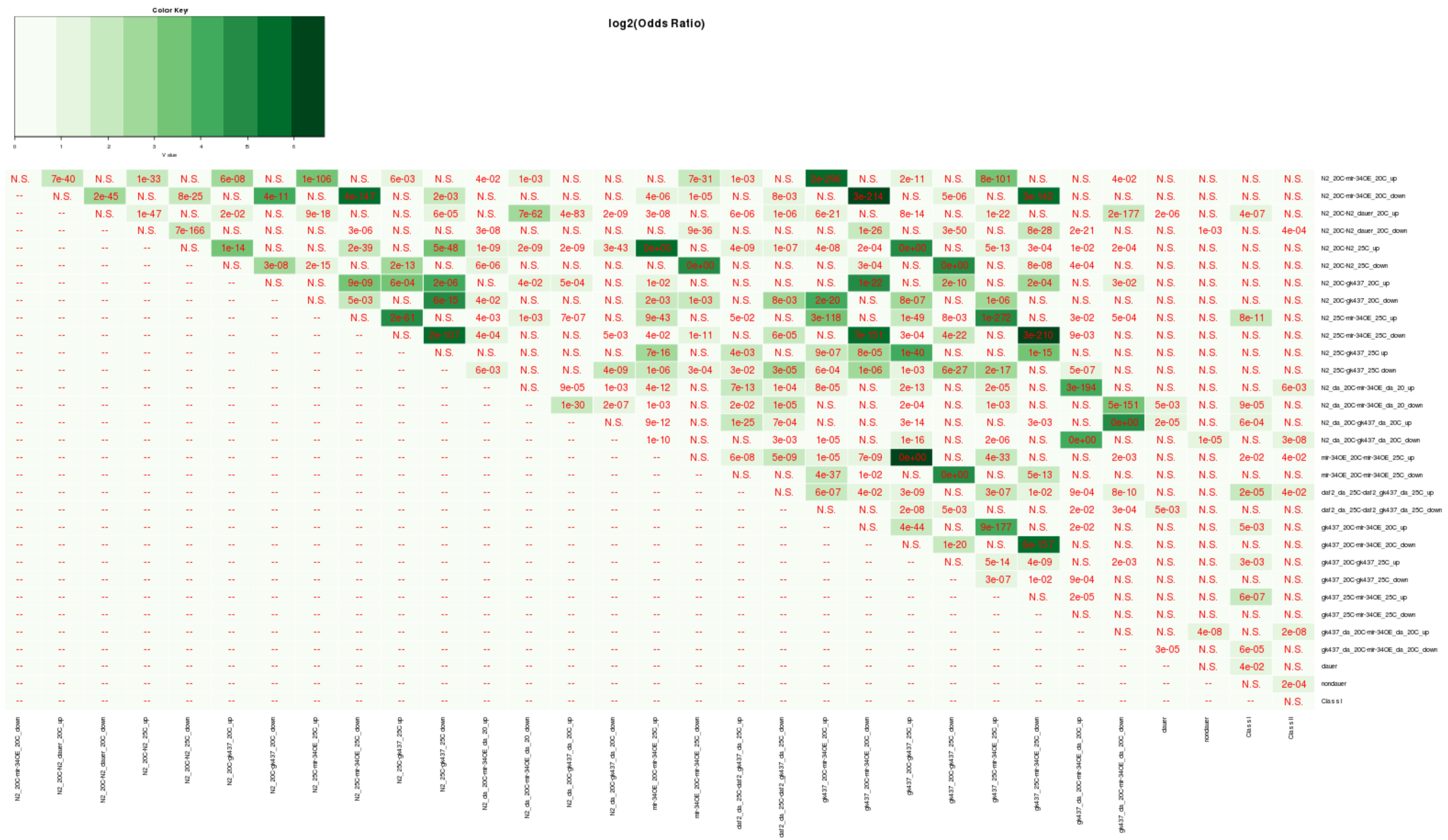


**Supplementary Figure S4. Gene overlap analysis for up- and down-regulated genes for conditions tested in microarray analysis, class 1, class 2, dauer and non-dauer genes.** GeneOverlap package was used to determine $log_2$(odds) ratio values (green scale). Numbers represent *P* values for statistical significance of overlaps (Fisher's exact test).

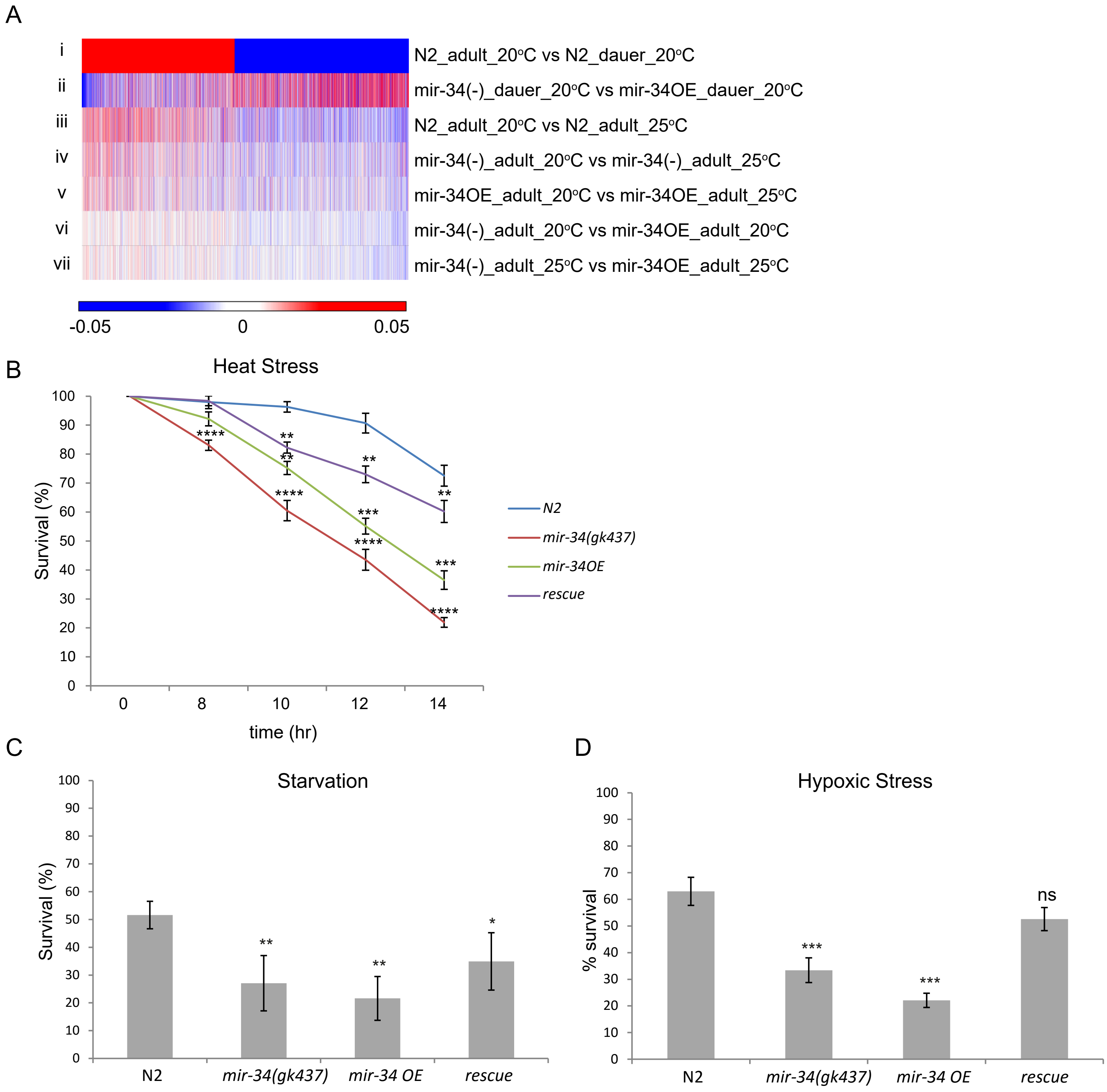


**Supplementary Figure S5. Both *mir-34(gk437)* and *mir-34OE* result in impaired stress response.** (**A**) Heatmap showing overlap between stress response genes induced by growth at high temperature and dauer formation and its correlation with *mir-34* mutation and overexpression in dauer and adult stages. (**B – D**) Survival under different stress conditions: heat stress (**B**), starvation (**C**) and hypoxic stress (**D**). Error bars represent SD. ***P* < 0.01, ***P* < 0.001, *****P* < 0.0001, unpaired two-tailed *t* test. All comparisons are to WT.

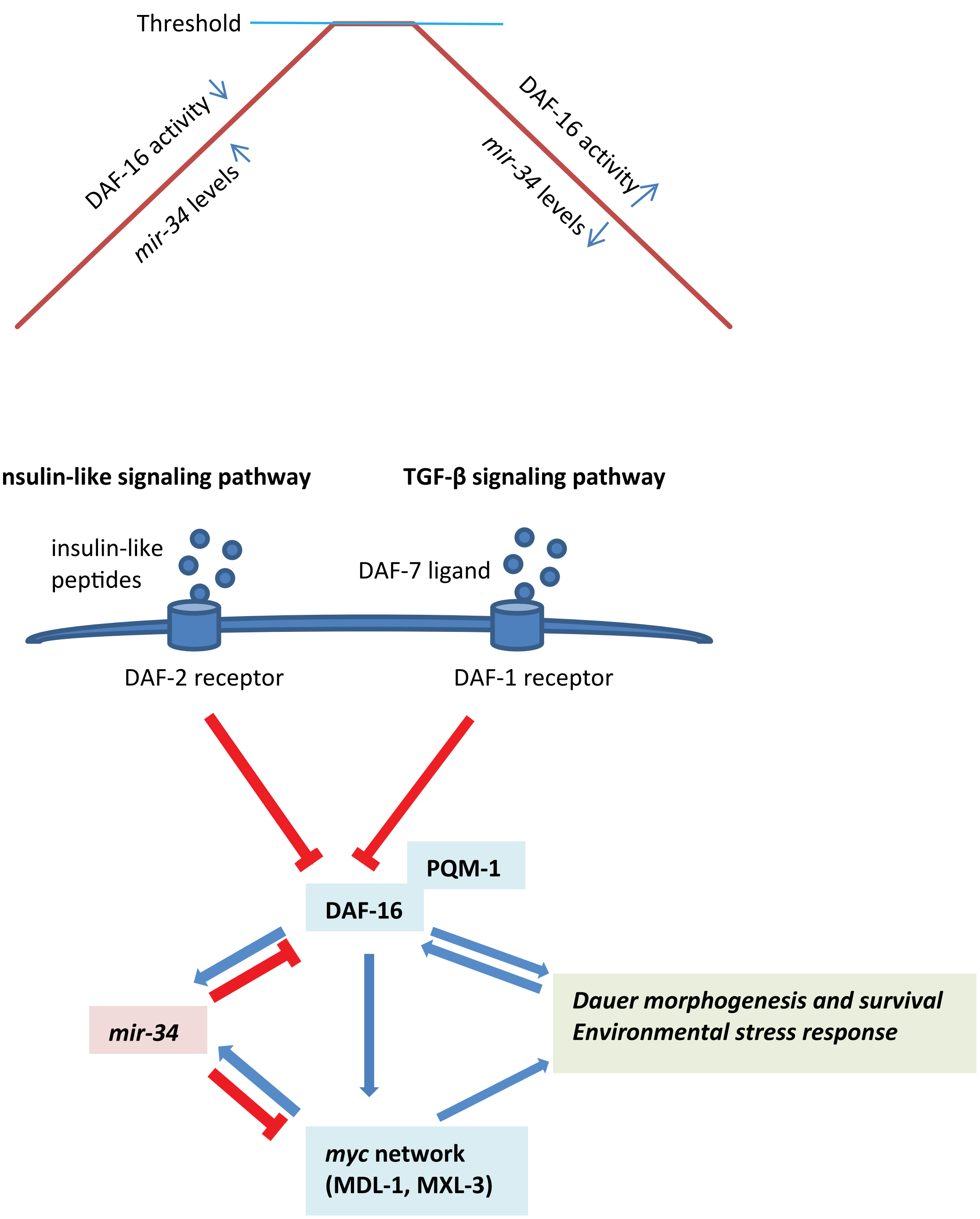


**Supplementary Figure S6. Model for *daf-16*/*mir-34* feedback inhibition loop in regulating dauer morphogenesis and survival and environmental stress response.** Insulin signaling pathway and other pathways (i.e TGF-β signaling pathway) that directly and indirectly regulate DAF-16 nuclear localization, respectively, can modulate *mir-34* levels. After *mir-34* levels reach a threshold, DAF-16 levels drop down via targeting of *daf-16* mRNA by miR-34. A similar feedback inhibition loop may also be present between *mir-34* and *myc* network. This crosstalk between *mir-34*, DAF-16 and *myc* network is necessary for regulation of dauer morphogenesis and survival and environmental stress response.

**Supplementary Table S4.** Strains used in this study.

| **Strain name** | **Genotype** |
|---|---|
| *BER107* | *mir-34(gk437)X (outcrossed 9x from VC1051)* |
| *BER108* | *daf-2(e1370)III (outcrossed 4x from CB1370)* |
| *BER109* | *daf-7(e1372)III (outcrossed 4x from CB1372)* |
| *BER110* | *daf-16(mu86)I (outcrossed 4x from CF1038* |
| *BER111* | *daf-16(mu86) I; muIs61 (outcrossed 9x from CF1139)* |
| *BER128* | *daf-16(mu86) I; muIs61; mir-34(gk437)* |
| *BER126* | *daf-2 (e1370)III;mir-34(gk437)* |
| *BER127* | *daf-7(e1372)III; mir-34(gk437)* |
| *BER124* | *unc-119(ed3)III oxIs[Pmir-34$_{2.2kb}$::mir-34; unc-119(+)]* |
| *BER125* | *unc-119(ed3)III oxIs[Pmir-34$_{2.2kb}$::mir-34; unc-119(+)]; mir-34(gk437)* |
| *BER101* | *unc-119(ed3)III Is[Pmir-34$_{2.2kb}$::gfp; unc-119(+)]* |
| *BER103* | *unc-119(ed3)III Is[Pmir-34$_{1.7kb}$::gfp; unc-119(+)]* |
| *BER102* | *unc-119(ed3)III Is[Pmir-34$_{1.2kb}$::gfp; unc-119(+)]* |
| *BER104* | *unc-119(ed3)III Is[Pmir-34$_{0.5kb}$:gfp; unc-119(+)]* |
| *BER105* | *unc-119(ed3)III Is[Pmir-34$_{\Delta seq1}$::gfp; unc-119(+)]* |
| *BER106* | *unc-119(ed3)III Is[Pmir-34$_{\Delta seq2}$::gfp; unc-119(+)]* |
| *BER113* | *daf-1(e1487)IV; unc-119(ed3)III Is[Pmir-34$_{2.2kb}$::gfp; unc-119(+)]* |
| *BER114* | *daf-2 (e1370)III; unc-119(ed3)III Is[Pmir-34$_{2.2kb}$::gfp; unc-119(+)]* |
| *BER115* | *daf-3(mgDf90)X; unc-119(ed3)III Is[Pmir-34$_{2.2kb}$::gfp; unc-119(+)]* |
| *BER116* | *daf-7(e1372)III; unc-119(ed3)III Is[Pmir-34$_{2.2kb}$::gfp; unc-119(+)]* |
| *BER117* | *daf-9(e1406)X; unc-119(ed3)III Is[Pmir-34$_{2.2kb}$::gfp; unc-119(+)]* |
| *BER120* | *daf-16(mu86)I; unc-119(ed3)III Is[Pmir-34$_{2.2kb}$::gfp; unc-119(+)]* |
| *BER121* | *daf-2(e1370)III; daf-16(mu86); unc-119(ed3)III Is[Pmir-34$_{2.2kb}$::gfp; unc-119(+)]* |
| *BER122* | *daf-7(e1372)III; daf-16(mu86); unc-119(ed3)III Is[Pmir-34$_{2.2kb}$::gfp; unc-119(+)]* |

**Supplementary Table S5.** Primer sequences used in this study

**Cloning of *mir-34* promoter**

| | |
|---|---|
| Pmir-34$_{2.2kb}$-PstI-F | gaactgcagccactggtttgcaaataattag |
| Pmir-34$_{2.2kb}$-XbaI-R | cgctctagacgttataagaataatagtcagtag |

**Cloning of *mir-34* gene**

| | |
|---|---|
| *mir-34-NotI-F* | atagcggccgcccactggtttgcaaataattag |
| *mir-34-BamHI-R* | ataGGATCCtcaaagaagcgttttaaagaag |

***mir-34* promoter truncations**

| | |
|---|---|
| *Pmir-34-Not1-R* | atagcggccgcCGTTATAAGAATAATAGTCAG |
| *Pmir-34$_{1.7kb}$-BsptI-F* | atacttaagGTTTGAGTTTAAAAAAAAAG |
| *Pmir-34$_{1.2kb}$-BsptI-F* | atacttaagAGCGAAGAGGGAGGTAAGGT |
| *Pmir-34$_{0.5kb}$-BsptI-F* | atacttaagTTCTTAGTAGTAGAAGAAGAA |

***mir-34* promoter deletions by Quickchange**

| | |
|---|---|
| *Pmir-34$_{\Delta seq2}$-F* | GAAAAGGAGCgggaaacatagatagaggtaAAAGGGAGTAAGAGGACAGGAACAgggtga |
| *Pmir-34$_{\Delta seq2}$-R* | tcacccTGTTCCTGTCCTCTTACTCCCTTTtacctctatctatgtttcccGCTCCTTTTC |
| *Pmir-34$_{\Delta seq1}$-F* | ggACGACAAGATAGATCGAAAAGGAGCACAGGAACAgggtgagaaccccgcct |
| *Pmir-34$_{\Delta seq1}$-R* | aggcggggttctcacccTGTTCCTGTGCTCCTTTTCGATCTATCTTGTCGTcc |

**Identification of *mir-34* mutation**

| | |
|---|---|
| *mir-34(gk437)-F1* | gaagatactcaaactttgcttg |
| *mir-34(gk437)-R1* | gaattcttgatcaatccattg |
| *mir-34(gk437)-F2* | gcctcgttcgttcgctcttg |
| *mir-34(gk437)-R2* | gaagcgttttaaagaagcgtcg |
